# Pulmonary Infections with Nontuberculous Mycobacteria, Catalonia, Spain, 1994–2014

**DOI:** 10.3201/eid2406.172095

**Published:** 2018-06

**Authors:** Miguel Santin, Irene Barrabeig, Pierre Malchair, Lucia Gonzalez-Luquero, Miguel A. Benitez, Josefina Sabria, Merce Palau-Benavent, Concepcion Cañete, Joan A. Lloret-Queraltó, Maria D. Grijota-Camino, Jordi Dorca, Fernando Alcaide

**Affiliations:** Bellvitge University Hospital-IDIBELL, L’Hospitalet de Llobregat, Spain (M. Santin, P. Malchair, L. Gonzalez-Luquero, M.D. Grijota-Camino, J. Dorca, F. Alcaide);; University of Barcelona, Barcelona, Spain (M. Santin, J. Dorca, F. Alcaide);; Agency of Public Health of Catalonia, Barcelona (I. Barrabeig);; Consorci del Laboratory Intercomarcal de l’Alt Penedès, l’Anoia i el Garraf, Vilafranca del Penedès, Spain (M.A. Benitez);; Hospital Moisés Broggi, Sant Joan Despí, Spain (J. Sabria, C. Cañete);; Hospital de Viladecans, Viladecans, Spain (M. Palau-Benavent, J.A. Lloret-Queraltó)

**Keywords:** nontuberculous mycobacteria, pulmonary infections, prevalence, tuberculosis and other mycobacteria, Catalonia, Spain, bacteria, respiratory infections

## Abstract

In Spain, systematic reporting of pulmonary infections with nontuberculous mycobacteria is not mandatory. Therefore, to determine trends, we retrospectively identified cases for January 1994–December 2014 in Catalonia. Over the 21 years, prevalence increased and was associated with being male. *Mycobacterium avium* complex and *M. abscessus* prevalence increased; *M. kansasii* prevalence decreased.

Nontuberculous mycobacteria (NTM) in low-prevalence settings have been regarded as opportunistic pathogens associated with HIV infection and chronic pulmonary disease. An overall increase in prevalence of pulmonary NTM infections in different geographic areas has been reported (*1*–*7*). The burden of pulmonary NTM infections in Spain is largely unknown because systematic reporting is not mandatory. We report secular trends in the prevalence of pulmonary infections caused by NTM over a 21-year period in a healthcare region of Catalonia, Spain.

## The Study

We performed a descriptive population-based study in 13 municipalities in the Barcelona-South Health Region of Catalonia for the period January 1994–December 2014. The study population comprised inhabitants >18 years of age in this area of the health region (population at the period midpoint 600,892). During 1994–2009, specialized healthcare for this population was provided by 3 hospitals and from 2010 on by 4 hospitals. The referral hospital for the area is Bellvitge University Hospital (BUH). Throughout the study period, the BUH mycobacterial laboratory processed all samples from the participating hospitals for identification only or for culture and identification. Samples for mycobacteria isolation were processed according to standard methods (*8*). From April 1994 through March 2009, liquid cultures were processed by use of the BACTEC TB 460 radiometric method (Becton Dickinson, Sparks, MD, USA); from April 2009 on, the BACTEC MGIT 960 system (Becton Dickinson) was used. From 1994 through 2002, *Mycobacterium abscessus* was part of the *M. chelonae-abscessus* group; from 2003 on, it was identified as a separate species.

Using files of NTM isolates from the BUH laboratory, we retrospectively identified cases and included only those for which patients were >18 years of age, resided in the study area, and had NTM isolated from >1 respiratory specimen. We excluded patients who resided outside the study area and patients from whom *M. gordonae* was isolated. We defined patients as having pulmonary disease if >2 cultures were positive or if antimicrobial chemotherapy considered active against the species isolated had been started. The BUH ethics committee approved the study.

We calculated overall and annual prevalence rates with 95% CIs as the number of patients with >1 isolate (isolation prevalence) and the number of patients with pulmonary disease (disease prevalence), divided by the population, according to the official census of Catalonia (https://www.idescat.cat/territori/?geomun&langen). We used Poisson regression models to estimate rate ratios of isolates and pulmonary disease caused by NTM per year. We constructed regression models for age groups 18–49, 50–65, and >65 years; to determine the relative prevalence of respiratory specimens, we adjusted all estimates for sex and year of isolation. Results of the regression models are expressed as relative risk with 95% CIs. We performed analyses by using SPSS version 18.0 for Windows (https://www.ibm.com/analytics/data-science/predictive-analytics/spss-statistical-software) and Epidat (https://www.sergas.es/Saude-publica/EPIDAT?idioma=es) statistical packages.

**Figure 1:**
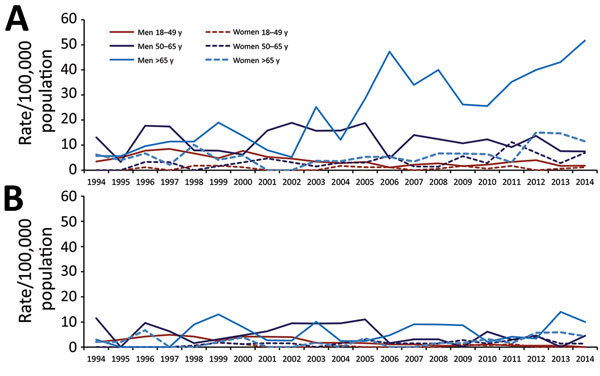
Annual prevalence rates per 100,000 population for nontuberculous mycobacteria isolation (A) and pulmonary disease (B), by patient sex and age group, Barcelona-South Health Region, Catalonia, Spain, 1994–2014.

During the 21-year period, we identified 680 patients (mean age 60.5 ± 16.6 years; 77.8% men) from whom NTM had been isolated from respiratory specimens. The overall period prevalence of patients from whom NTM was isolated was 113.2 (95% CI 105.0–122.0)/100,000 population; for pulmonary disease, prevalence was 42.8 (95% CI 37.5–48.0)/100,000 population. Prevalence rates were higher for men than for women, for isolation (180.5 and 49.1/100,000 population, respectively), and for disease (68.2 and 18.5/100,000 population, respectively) (Figure 1; Technical Appendix Tables 1, 2). The regression models showed a 5% annual decrease in prevalence rates of isolation among those 18–49 years of age, an 11% increase among those >65 years of age, and no significant changes among those 50–65 years of age. Male sex remained an independent factor associated with higher rates of isolation of NTM in the 3 age groups (Table 1; Figure 1, panel A). Similarly, prevalence rates of pulmonary disease showed an annual decrease of 9% among those 18–49 years of age, an annual increase of 7% among those >65 years of age, and remained without significant changes among those 50–65 years of age. Male sex remained associated with higher rates of pulmonary disease (Table 1; Figure 1, panel B).

**Table 1:** Poisson regression analysis of the association between prevalence rates of nontuberculous mycobacteria isolation and pulmonary disease, Barcelona-South Health Region, Catalonia, Spain, 1994–2014*

Patient age group, sex, and year	Isolation				Pulmonary disease		
	No. patients	aRR (95% CI)	p value		No. patients	aRR (95% CI)	p value
18–49 y	176				89		
Sex							
F	29	1.0	<0.0001		12	1.0	<0.0001
M	147	5.10 (3.20–8.12)			77	6.46 (3.04–13.89)	
Year of isolation	176	0.95 (0.93–0.97)	<0.0001		89	0.92 (0.88–0.95)	<0.0001
50–65 y	209				89		
Sex							
F	49	1.0	<0.0001		20	1.0	<0.0001
M	160	3.18 (2.20–4.58)			69	3.38 (2.01–5.24)	
Year of isolation	209	1.02 (0.99–1.04)	0.2		89	0.99 (0.95–1.04)	0.7
>65 y	295				79		
Sex							
F	73	1.0	<0.0001		25	1.0	0.001
M	222	3.09 (2.39–3.93)			54	2.15 (1.35–3.41)	
Year of isolation	295	1.11 (1.09–1.13)	<0.0001		79	1.0 (1.02–1.12)	0.004

*aRR, adjusted relative risk.

**Figure 2:**
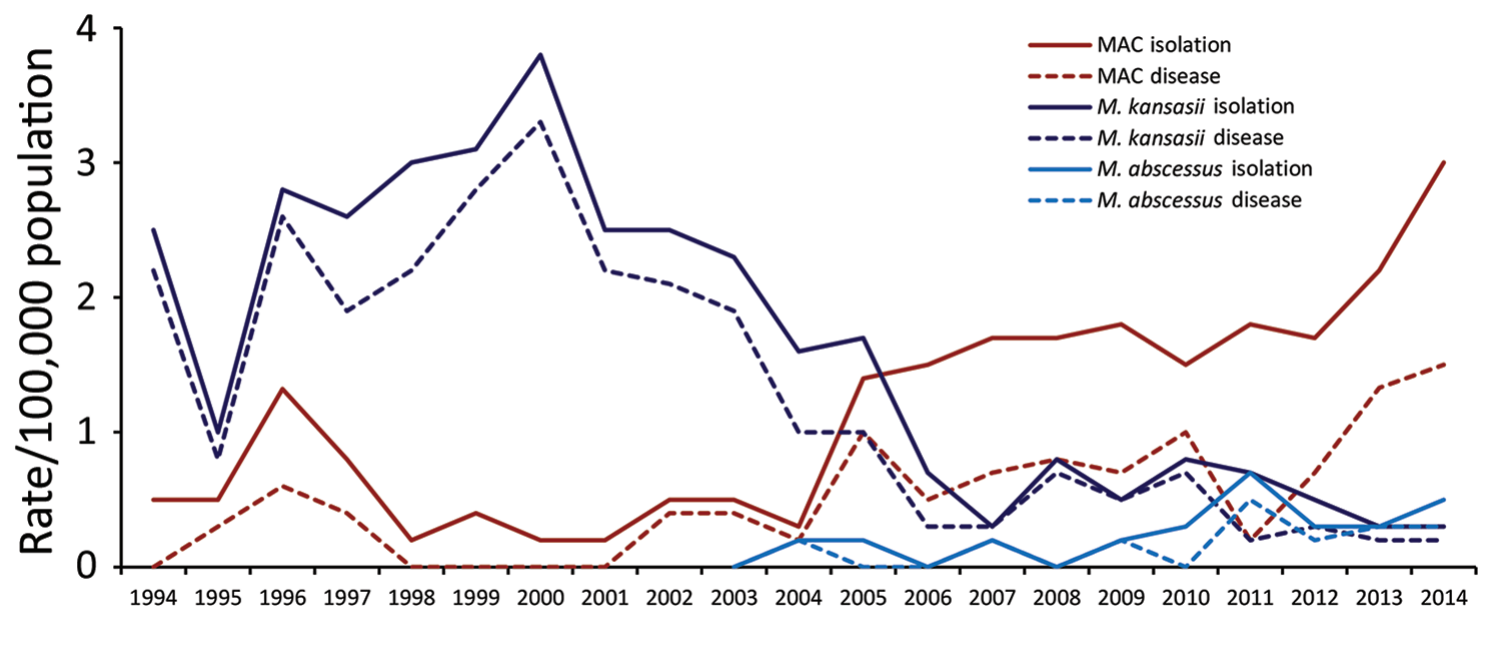
Annual prevalence rates per 100,000 population of 3 species of nontuberculous mycobacteria (*Mycobacterium kansasii*, MAC, and *M. abscessus*), Barcelona-South Health Region, Catalonia, Spain, 1994–2014. MAC, *Mycobacterium avium* complex.

Trends in prevalence rates differed among species of mycobacteria (Table 2; Figure 2). Although prevalence rates rose significantly for *M. avium* complex (MAC) (by 10% for isolation and 13% for pulmonary disease), rates of *M. kansasii* fell (by 9% for isolation and 11% for pulmonary disease). Since 2003, *M. abscessus* isolation increased annually by 22% and pulmonary disease increased by 24%. As for rapidly growing mycobacteria other than *M. abscessus*, isolation but not pulmonary disease increased significantly over the study period (Table 2).

**Table 2:** Bivariate Poisson regression analysis of changes in prevalence rates of nontuberculous mycobacteria isolation and pulmonary disease by species. Barcelona-South Health Region, Catalonia, Spain, 1994–2014*

Species	Isolation				Pulmonary disease		
	No. patients	RR (95% CI)	p value		No. patients	RR (95% CI)	p value
*Mycobacterium kansasii*	194	0.92 (0.89–0.94)	<0.0001		154	0.91 (0.87–0.93)	<0.0001
*M. avium* complex	139	1.10 (1.07–1.15)	<0.0001		67	1.13 (1.08–1.18)	<0.0001
*M. xenopi*	96	1.03 (0.99–1.08)	0.16		16	1.06 (0.98–1.13)	0.10
*M. abscessus*†	17	1.22 (1.11–1.33)	<0.0001		11	1.24 (1.08.1.42)	0.002
Other‡	234	1.10 (1.08–1.12)	<0.0001		9	1.11 (0.99–1.24)	0.06

*RR, relative risk †2003–2014 only. ‡Rapidly growing mycobacteria other than *M. abscessus,* and other species of mycobacteria.

## Conclusions

We found a significant increase in the prevalence of isolation of NTM from respiratory specimens in the study area. However, the trends varied according to mycobacteria species. The most common NTM causing lung disease during the first half of the study period, *M. kansasii*, declined progressively from the early 2000s on. In contrast, MAC became the most frequently isolated NTM, and rates increased for *M. abscessus*, eventually equaling those of *M. kansasii*. Rapidly growing mycobacteria other than *M. abscessus* were also increasingly isolated in the most recent years, but most were deemed colonizers (*9*).

We do not believe that the increased prevalence of most NTM can be explained by the 2009 implementation of the new culture system MGIT 960 because its sensitivity is equivalent to that of the BACTEC TB 460 system used previously in combination with solid media (*10*,*11*). Furthermore, changes in MAC and *M. kansasii,* which are easily recovered in culture, cannot be attributed to the implementation of the newer culture media. The changes recorded in temporal trends can be explained, at least in part, by the dynamics of the HIV epidemic in Spain. The reduction of susceptible HIV-infected patients through improvements in antiretroviral therapy in the second half of the 1990s led to a dramatic fall in the prevalence of NTM infections among these patients. Because *M. kansasii* was the most frequently isolated NTM among HIV-infected patients in the Barcelona-South Health Region of Catalonia, its prevalence decreased, paralleling the decrease in susceptible persons with HIV infection (*12*). This reduction in *M. kansasii* infections is also illustrated by the reduction among persons 18–49 years of age because HIV-infected patients in Spain at that time were mostly young users of illicit intravenous drugs. Even so, the decrease among HIV patients does not explain the changes in the NTM ecology in our area, in which MAC and *M. abscessus* seem to be filling the vacancy left by *M. kansasii*. Although the prevalence is still low, the current distribution of mycobacteria species in this area is closer to that of other countries (*1*–*7*,*13*).

Our study has 2 main limitations. First, although we aimed to capture all NTM patients in the area, we may have missed some patients who sought medical care elsewhere. Second, we defined pulmonary disease patients as having had >2 positive cultures or having received treatment for the NTM species isolated. Although the first criterion may have overestimated prevalence, the second may have been biased toward inclusion of patients with more severe illness and may thus have underestimated prevalence.

Our data add evidence for the increasing prevalence of NTM pulmonary infections in Catalonia. We also observed concurrent species-specific differences in prevalence trends in this area.

Technical Appendix Annual prevalence rates for nontuberculous mycobacteria isolation, pulmonary disease, and 3 species of nontuberculous mycobacteria, Barcelona-South Health Region of Catalonia, Spain, 1994–2014.
