# Supplementary material for: Pulmonary Infections with Nontuberculous Mycobacteria, Catalonia, Spain, 1994–2014
Source: Emerg Infect Dis. 2018 Jun;24(6):1091–4. doi: 10.3201/eid2406.172095 (PMC6004863; doi:10.3201/eid2406.172095)
Supplement: Technical Appendix — Annual prevalence rates for nontuberculous mycobacteria isolation, pulmonary disease, and 3 species of nontuberculous mycobacteria, Barcelona-South Health Region of Catalonia, Spain, 1994–2014. [file 17-2095-Techapp-s1.pdf]

Article DOI: https://doi.org/10.3201/eid2406.172095

Pulmonary Infections with Nontuberculous Mycobacteria, Catalonia, Spain, 1994–2014

Technical Appendix

Technical Appendix Table 1. Prevalence rates of patients with isolation and pulmonary disease of nontuberculous mycobacteria by age group, Barcelona-South Health Region of Catalonia, 1994–2014

	Isolation		Pulmonary disease	
Patient age group, sex, and year	No. patients	Prevalence ratio/100,000 (95% CI)*	No. patients	Prevalence ratio/100,000 (95% CI)*
18–49 y	176		89	
Sex				
F	29	15.9 (10.1–21.7)	12	0.6 (2.8–10.3)
M	147	79.1 (66.3–91.8)	77	641.4 (32.2–50.6)
Year of isolation	176	47.7 (40.7–54.8)	89	24.1 (19.1–29.2)
50–65 y	209		89	
Sex				
F	49	74.0 (53.3–94.7)	20	30.2 (17.0–43.4)
M	160	251.7 (213.0–291.0)	69	108.5 (82.9–134.0)
Year of isolation	209	161.1 (139.0–183.0)	89	68.6 (54.3–82.8)
>65 y	295		79	
Sex				
F	73	123.8 (95.4–152.0)	25	42.4 (25.8–59.0)
M	222	509.9 (443.0–577.0)	54	124.0 (91.0–157.0)
Year of isolation	295	287.8 (255.0–321.0)	79	77.1 (60.1–94.1)

*Overall period prevalence.

Technical Appendix Table 2. Prevalence rates of patients with nontuberculous mycobacteria by species. Barcelona-South Health Region of Catalonia, 1994–2014

	Isolation		Pulmonary disease	
Species	No. patients	Prevalence ratio/100,000 (95% CI)*	No. patients	Prevalence ratio/100,000 (95% CI)*
Mycobacterium kansasii	194	32.3 (27.7–36.8)	154	25.6 (21.6–29.7)
Mycobacterium avium complex	139	23.1 (19.3–27.0)	67	11.2 (8.5–13.8)
Mycobacterium xenopi	96	15.9 (12.0–19.2)	16	2.66 (1.4–3.9)
Mycobacterium abscessus	17	2.83 (1.5–4.2)	11	1.83 (0.7–2.9)
Other†	234	38.9 (34.0–43.9)	9	1.5 (0.5–2.5)

*Overall period prevalence.
†Rapidly growing mycobacteria other than *Mycobacterium abscessus*, and other mycobacteria species.

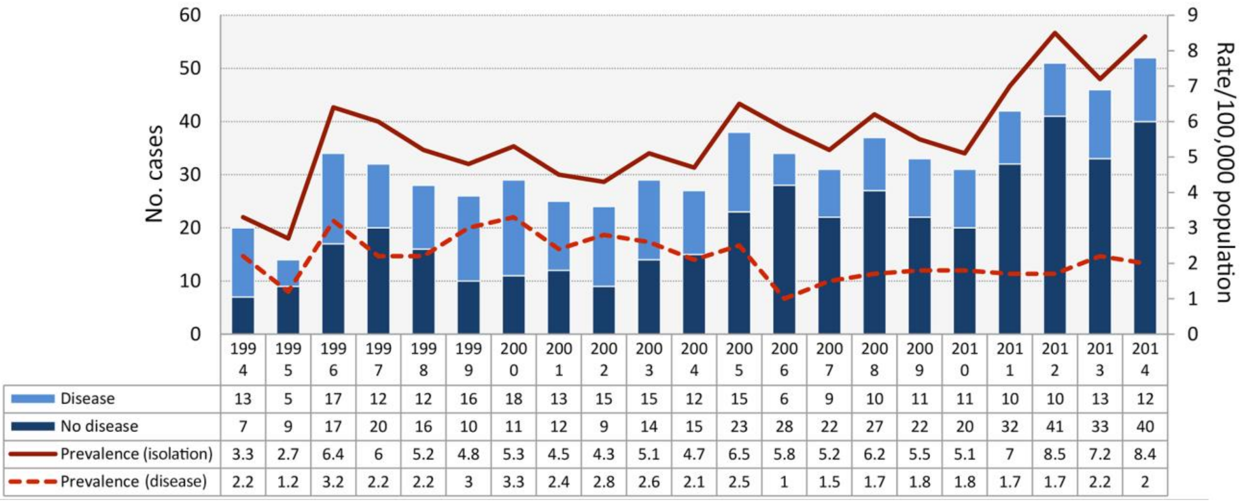

	1994	1995	1996	1997	1998	1999	2000	2001	2002	2003	2004	2005	2006	2007	2008	2009	2010	2011	2012	2013	2014
Disease	13	5	17	12	12	16	18	13	15	15	12	15	6	9	10	11	11	10	10	13	12
No disease	7	9	17	20	16	10	11	12	9	14	15	23	28	22	27	22	20	32	41	33	40
Prevalence (isolation)	3.3	2.7	6.4	6	5.2	4.8	5.3	4.5	4.3	5.1	4.7	6.5	5.8	5.2	6.2	5.5	5.1	7	8.5	7.2	8.4
Prevalence (disease)	2.2	1.2	3.2	2.2	2.2	3	3.3	2.4	2.8	2.6	2.1	2.5	1	1.5	1.7	1.8	1.8	1.7	1.7	2.2	2

Technical Appendix Figure. Trends in isolation and pulmonary disease of nontuberculous mycobacteria from respiratory specimens over a 21-year period, Barcelona-South Health Region of Catalonia, 1994–2014.
